# Automating iPSC generation to enable autologous photoreceptor cell replacement therapy

**DOI:** 10.1186/s12967-023-03966-2

**Published:** 2023-02-28

**Authors:** Laura R. Bohrer, Nicholas E. Stone, Nathaniel K. Mullin, Andrew P. Voigt, Kristin R. Anfinson, Jessica L. Fick, Viviane Luangphakdy, Bradley Hittle, Kimerly Powell, George F. Muschler, Robert F. Mullins, Edwin M. Stone, Budd A. Tucker

**Affiliations:** 1 Institute for Vision Research, Carver College of Medicine, University of Iowa, 375 Newton Road, Iowa City, IA 52242 USA; 2 Department of Ophthalmology and Visual Sciences, Carver College of Medicine, University of Iowa, Iowa City, IA USA; 3 Department of Biomedical Informatics, The Ohio State University, Columbus, OH USA; 4 Department of Biomedical Engineering, Lerner Research Institute, Cleveland Clinic, Cleveland, OH USA; 5 Department of Orthopaedic Surgery, Cleveland Clinic, Cleveland, OH USA; 6 Cell X Technologies Inc, Cleveland, OH USA

**Keywords:** Induced pluripotent stem cells, RNA sequencing, Retinal differentiation, Robotic cell culture, Automation, Cell therapy, Cell manufacturing

## Abstract

**Background:**

Inherited retinal degeneration is a leading cause of incurable vision loss in the developed world. While autologous iPSC mediated photoreceptor cell replacement is theoretically possible, the lack of commercially available technologies designed to enable high throughput parallel production of patient specific therapeutics has hindered clinical translation.

**Methods:**

In this study, we describe the use of the Cell X precision robotic cell culture platform to enable parallel production of clinical grade patient specific iPSCs. The Cell X is housed within an ISO Class 5 cGMP compliant closed aseptic isolator (Biospherix XVivo X2), where all procedures from fibroblast culture to iPSC generation, clonal expansion and retinal differentiation were performed.

**Results:**

Patient iPSCs generated using the Cell X platform were determined to be pluripotent via score card analysis and genetically stable via karyotyping. As determined via immunostaining and confocal microscopy, iPSCs generated using the Cell X platform gave rise to retinal organoids that were indistinguishable from organoids derived from manually generated iPSCs. In addition, at 120 days post-differentiation, single-cell RNA sequencing analysis revealed that cells generated using the Cell X platform were comparable to those generated under manual conditions in a separate laboratory.

**Conclusion:**

We have successfully developed a robotic iPSC generation platform and standard operating procedures for production of high-quality photoreceptor precursor cells that are compatible with current good manufacturing practices. This system will enable clinical grade production of iPSCs for autologous retinal cell replacement.

**Supplementary Information:**

The online version contains supplementary material available at 10.1186/s12967-023-03966-2.

## Background

Since the first successful kidney transplant between identical twins in 1954 [1], the field of regenerative medicine has flourished. Transplantation of HLA matched solid organs is now commonplace, saving thousands of lives each year [2, 3]. The success of solid organ transplantation can in part be credited to the fact that the tissue remains viable for an extended period post-harvest and that it is possible to reestablish functional connections with the host via a combination of microsurgery and peripheral nervous system (PNS) reinnervation. Unlike peripheral organs, mature tissues of the central nervous system (CNS) are not afforded the same advantages. Specifically, CNS tissues sustain irreversible damage within minutes after perfusion is lost. Furthermore, due to both environmental and cell intrinsic properties, the ability of mature CNS neurons to regenerate is limited. For instance, following subretinal transplantation into retinal degenerative mice, intact sheets of mature photoreceptor cells fail to extend axons or make synaptic connections with host retinal interneurons [4]. In contrast, developing retinal progenitor cells can readily integrate with the dystrophic host retina post-transplantation [5]. For this reason, we and others have focused our attention on developing stem cell-based photoreceptor cell replacement strategies for the treatment of patients with retinal degenerative blindness [6–23].

While retinal progenitor and postmitotic photoreceptor precursor cells are at an appropriate stage of development for retinal transplantation [5, 24–27], these cells can only be harvested from late-stage fetal donor retina making them both ethically unfavorable and difficult to obtain in sufficient numbers to be clinically viable. Rather than isolate retinal progenitor cells from a developing fetus, the field has focused much of its attention on the development of protocols designed to guide differentiation of pluripotent stem cells into the desired cell types [28–44].

Both embryonic (ESCs) and induced pluripotent stem cells (iPSCs) have been used to generate transplantable retinal progenitor and photoreceptor precursor cells [8–18, 20, 21, 44]. While ESCs are not devoid of ethical debate, the National Institutes of Health has maintained a registry of established ES cell lines that are acceptable for use in NIH funded work [45]. These lines have been fully characterized and are readily available for distribution. The advantage of using a single ESC line for clinical cell replacement is that a validated master cell bank of transplantable progeny can be created using readily available large scale manufacturing approaches. A drawback associated with this approach is that the donor and recipient are genetically discordant (i.e., allogenic). To ensure allograft longevity, lifelong immune suppression will likely be required.

Unlike ES cells, iPSCs can be generated from the patient in need (i.e., autologous), greatly reducing the chance of immune rejection and need for lifelong immune suppression following transplantation [46, 47]. Unfortunately, current manufacturing strategies, which are primarily designed for large scale manufacturing of a single product, are not well suited for production of autologous cell therapies. Autologous photoreceptor cell replacement will require production of patient-derived iPSCs and derivation of retinal progenitor cells from dozens of individuals in parallel. While this could be done by simply adding technical staff, each of whom are responsible for generating a small number of cell lines under current good manufacturing practices (cGMP), this strategy would dramatically increase production costs and result in greater product variability. To address these issues, robotic platforms that can automate the labor-intensive steps required for iPSC-based therapeutic manufacturing are needed.

In this manuscript we describe the successful generation of clinical grade patient-specific iPSCs using the Cell X™ precision robotic platform [48]. This platform was designed to perform many of the critical steps associated with autologous cell line production, including imaging, media exchange, picking, weeding and clonal expansion of iPSCs. By placing the Cell X platform inside of a cGMP-compliant Biospherix Xvivo system, we have been able to reliably generate high quality patient iPSCs that retain a normal karyotype, are pluripotent, and can differentiate into retinal organoids containing transplantable retinal progenitor and photoreceptor precursor cells. Resulting iPSC-derived progeny were found to be indistinguishable from those generated using manual processing. In summary, incorporation of the Cell X platform into a clinical iPSC production pipeline will greatly enhance manufacturing throughput, enable product trackability, and reduce batch-to-batch variation associated with protocol drift, differences between technical staff, and human error.

## Methods

### Cellular reprogramming

Reprogramming, clonal expansion, and retinal differentiation were all performed using the Cell X™ platform (Cell X Technologies Inc, Cleveland, OH), which is contained within a cGMP compliant Biospherix Xvivo system (BioSpherix, Ltd., Parish, NY) using cGMP compliant reagents. Dermal fibroblasts isolated from skin biopsies obtained from patients with inherited retinal degenerative blindness were reprogrammed using the CytoTune2 kit (Thermo Fisher Scientific, Waltham, MA), a non-integrating Sendai viral reprogramming kit, as previously reported [49]. Briefly, fibroblasts isolated from a 3 mm dermal punch biopsy and cultured at 37 °C and 5% CO2 and 20% O2 in biopsy media [49]. For consistency, 250,000 fibroblasts are transduced on Monday in one well of a 6-well culture dish at a target MOI of 5 with Sendai viral vectors driving expression of OCT4, SOX2, KLF4 and C-MYC. Viral reprograming media is replaced on Tuesday and cultures are subsequently fed (i.e., media was changed) on Wednesday. On Friday, 4 days following transduction, cells were passaged onto a 6 well laminin 521 (LN521, BioLamina, Sundbyberg, Sweden) coated culture dish at 5000, 10,000, and 20,000 cells per well (i.e., 3 separate wells). At 24 h following passage, media was changed to complete Essential 8 (E8) medium (CTS, Thermo Fisher Scientific) and the oxygen tension was dropped to 10% where it was maintained until after colony picking. Each time the culture was fed (i.e., every Monday, Wednesday, and Friday), the entire surface of the culture plate was scanned using the Cell X™ platform, which allows for tracking of cells from fibroblast isolation through growth, picking, and clonal expansion of iPSCs. Once colonies reached sufficient size (~ 1.5–3 mm in diameter), the Cell X™ platform was used to pick 12 individual clones (selected from the larger population of existing colonies based on morphology (i.e., high nucleus to cytoplasm ratio), and growth characteristics (i.e., densely packed with clearly demarcated phase bright edge and little spontaneous differentiation)), which were each transferred into a separate well of a laminin 521 coated 12 well culture dish for expansion. After picking, the original culture was re-scanned to confirm that the desired cells had been successfully removed. Following the initial passage, O2 concentration was raised to 20%, iPSCs were fed daily and passaged onto laminin 521 coated culture dishes using Versene (CTS, Thermo Fisher Scientific) once they reached 80%. Except for moving cell culture dishes to and from the incubator (which is done by hand), all steps, including imaging, feeding and passage, are automated. To prevent Sendai virus contamination of the media dispensing tubing and head, fibroblast cell transduction was performed manually in the adjacent cell processing chamber. At passage 10, iPSC lines were subject to karyotyping and scorecard analysis to confirm genetic integrity and potency. To further validate this approach, iPSCs generated on the Cell X™ platform were compared to those generated manually using our previously published methods [49].

### Karyotype and ScoreCard™ analysis

IPSCs were karyotyped in metaphase by the Shivanand R. Patil Cytogenetics and Molecular Laboratory at the University of Iowa using Leica Microsystems metaphase scanning platform and CytoVision version 7.7 software. Cells were grown in vitro and arrested at metaphase with colcemid. Chromosomes were stained by the G-banding method, counted and structurally evaluated for the presence or absence of detectable rearrangements. At least 20 cells were analyzed for each iPSC line. For TaqMan hPSC ScoreCard™ analysis, total RNA was isolated using the NucleoSpin RNA purification kit (Takara Bio, San Jose, CA). cDNA was generated from 1 µg of RNA using VILO cDNA synthesis kit (Thermo Fisher Scientific). cDNA was added to a TaqMan hPSC scorecard plate (Thermo Fisher Scientific) and amplified using a QuantStudio 6 Flex real-time PCR system. Results were analyzed using Thermo Fisher’s cloud-based analysis suite.

### Retinal differentiation of iPSCs generated on cell X™ platform

Retinal differentiation was performed with modifications for GMP-compliance as described previously [31, 50]. IPSCs were cultured on laminin 521 coated plates in E8 medium. Embryoid bodies (EBs) were lifted with ReLeSR (STEMCELL Technologies, Cambridge, MA) and transitioned from E8 to neural induction medium (NIM- DMEM / F12 (1:1), 1% N2 supplement, 1% non-essential amino acids, 1% Glutamax (Thermo Fisher Scientific), 2 µg / mL heparin (Sigma-Aldrich, St. Louis MO) and Primocin (InvivoGen, San Diego, CA)) over a four-day period. On day 6, NIM was supplemented with 1.5 nM BMP4 (R&D Systems, Minneapolis, MN). On day 7, EBs were adhered to Maxgel coated plates (Sigma-Aldrich). BMP4 was gradually transitioned out of the NIM over seven days. On day 16, the media was changed to retinal differentiation medium (RDM - DMEM / F12 (3:1), 2% B27 supplement (Thermo Fisher Scientific), 1% non-essential amino acids, 1% Glutamax and 0.2% Primocin). On day 25–30 the entire EB outgrowth was mechanically lifted using a cell scraper and transferred to ultra-low attachment flasks in 3D-RDM (RDM plus 10% KnockOut serum replacement (KSR); Thermo Fisher Scientific), 100 µM taurine (Sigma-Aldrich), 1:1000 chemically defined lipid concentrate (Thermo Fisher Scientific), and 1 µM all-trans retinoic acid (until day 100; Sigma-Aldrich). The cells were fed three times per week with 3D-RDM until harvest.

### Immunocytochemistry

At Day 120 and 160, organoids were fixed with 4% paraformaldehyde for 30–60 min at room temperature and equilibrated to 15% sucrose in PBS, followed by 30% sucrose. Organoids were cryopreserved in 50:50 solution of 30% sucrose / PBS: tissue freezing medium (Electron Microscopy Sciences, Hatfield, PA) and cryosectioned (15 μm). Sections were blocked with 5% normal donkey serum, 3% bovine serum albumin, and 0.1% triton-x and stained overnight with the following primary antibodies: OTX2 (R&D Systems; Cat# AF1979), Recoverin (EMD Millipore, Burlington, MA; Cat# AB5585), NRL (R&D Systems; Cat# AF2945), ARR3 (Lifespan Biosciences, Seattle, WA; Cat# LS-C368677), ML-Opsin (EMD Millipore; Cat# AB5405) and NR2E3 (R&D Systems; Cat# PP-H7223-00). The following secondary antibodies (Thermo Fisher Scientific) were incubated for 1 h: donkey anti-goat 488 (Cat# R37118), donkey anti-mouse 488 (Cat# A21202), and donkey anti-rabbit 647 (Cat# A31573). Cell nuclei were counterstained using DAPI (Thermo Fisher Scientific; Cat# 62,248). Images were acquired using a Leica TCS SPE upright confocal microscope system (Leica Microsystems, Wetzlar, Germany).

### Organoid dissociation

Ten representative organoids were selected from culture. Organoids were allowed to settle by gravity in a 1.5 mL tube and medium was aspirated. Organoids were then dissociated with 20 units / mL of papain (Worthington Biochemical Corporation, Lakewood, NJ) with 60 units / mL DNase I (Worthington Biochemical Corporation) on a shaker at 37 ˚C for approximately 60 min. Following dissociation, cells were pelleted at 400 × *g* for 5 min, resuspended in dPBS -/- (Thermo Fisher Scientific) with 0.04% non-acetylated bovine serum albumin (New England Biolabs, Ipswich, MA), and processed immediately for encapsulation and barcoding using the Chromium Controller (10X Genomics, Pleasanton, CA).

### Single-cell RNA sequencing (scRNA-seq) library construction

Cells were filtered through a 70 µm filter and further diluted to target 8000 cells per run. Single cells were then partitioned and barcoded with the Chromium Controller instrument (10X Genomics) and Single Cell 3’ Reagent (v3.1 chemistry) kit (10X Genomics) according to the manufacturer’s specifications with no modification (Rev C). Final libraries were quantified using the Qubit dsDNA HS Assay Kit (Thermo Fisher Scientific) and diluted to 3 ng/µL in buffer EB (Qiagen, Hilden, Germany). Library quality and concentration was confirmed using the Bioanalyzer High Sensitivity DNA Assay (Agilent Technologies, Santa Clara, CA) prior to sequencing.

### Sequencing, preprocessing, and mapping

scRNA libraries were sequenced using the NovaSeq 6000 instrument (Illumina, San Diego, CA) generating 100-bp paired end reads. Sequencing was performed by the Genomics Division of the Iowa Institute of Human Genetics. FASTQ files were generated from base calls with the bcl2fastq software (Illumina), and reads were mapped to the pre-built GRCh38 reference with Cell Ranger (version 6.0) (10X Genomics) using the ‘count’ function. Only cells passing the default Cell Ranger call were analyzed further. Only cells with between 500 and 7,000 unique genes (features) and with < 15% of reads mapping to mtDNA-encoded genes and < 25% of reads mapping to ribosomal genes were included in the analysis.

### Computational analysis

Filtered libraries were normalized with Seurat [51]. Variable features were identified with the vst selection method before scaling and dimensionality reduction. To compare the newly generated data with existing studies, data from this manuscript were integrated with previously generated scRNA-seq data of retinal organoid development at day 90, 104, and 110 [52]. Integration was performed with canonical correlation analysis using the Seurat package (v3.2.3). To model maturation of progenitors into photoreceptors, cells identified as retinal progenitors, transitionary cells (T1 and T3 populations), and rods were subset from the original Seurat object. We applied the PHATE dimensionality reduction (v1.0.7) [53] to the integrated, scaled data from the photoreceptor and precursor subset population. A total of 10 nearest neighbors were identified for each cell, and these neighbors were used to build the kernel with a decay factor of 75. Next, the slingshot R package [54] was used to create a trajectory analysis using the PHATE embeddings, with the trajectory beginning at the progenitor population.

## Results

### Automated generation of patient-derived iPSCs

**Fig. 1:**
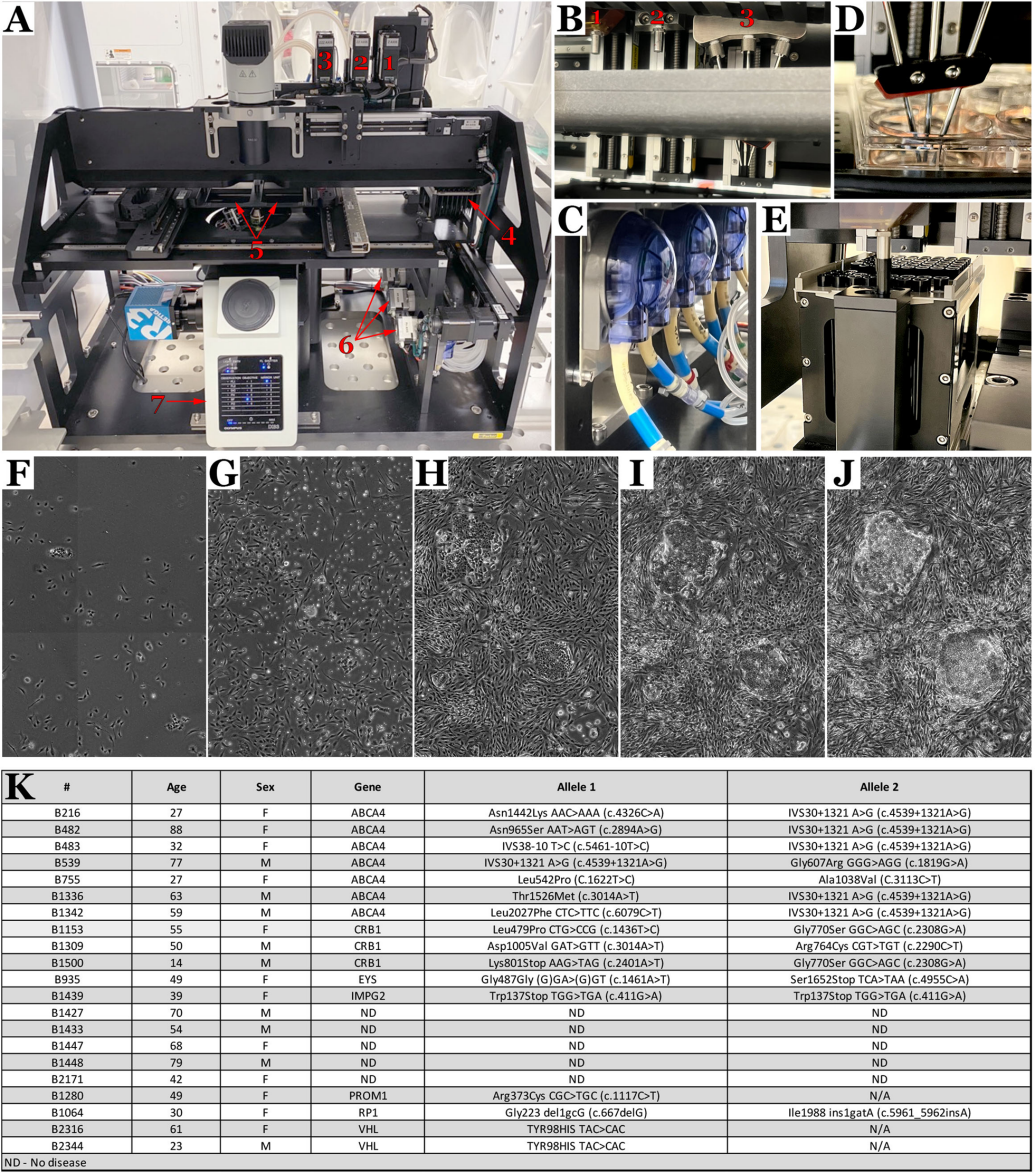
The Cell X Robotic platform for automated generation of iPSCs from primary dermal fibroblasts. **A** The Cell X platform consists of a robotic liquid handling system (**A1**–**A3**, & **A6**) mounted on a commercially available inverted fluorescent microscope (**A7**). Up to two standard multi-well plates can be placed on the system (**A5**), which is capable of imaging the cultures, performing media exchanges, weeding unwanted cells and picking iPSC colonies, which can then be transferred into different wells or plates. **A1** = syringe pump, **A2** = aspiration pump, **A3** = media dispense pump head, **A4** = disposable micro pipette tips for both the syringe and aspiration pumps, **A5** = robotic stage that holds two multi-well cell culture plates, A6 = peristaltic pumps which supply media to **A3**, **A7** = automated microscope. **B** The Cell X platform has robust liquid handling capabilities, enabled by a robotic syringe pump (**B1**) for picking and weeding, an aspiration pump (**B2**) for removing media as well as three dispensing pumps (**B3**) for adding culture media and other liquid reagents to individual wells. **C** Dispensing of liquid reagents and aspiration is accomplished using software controlled peristaltic pumps. **D** Three different liquid reagents can be placed into wells via separate dispensing needles. **E** Both the syringe and aspiration pump use disposable micropipette tips to ensure aseptic cell culture conditions and to prevent cross-contamination between samples. **F–J** The automated imaging capabilities of the Cell X platform enable the growth of individual iPSC colonies to be monitored over time. **K** During this study we have used the Cell X to generate 21 iPSC lines from dermal fibroblast samples obtained from both male and female patients between the ages of 14 and 88 who have disease causing mutations in several different genes

The Cell X platform (Fig. 1A) is a robotic imaging and liquid handling system capable of automating many of the labor-intensive procedures required for iPSC generation, while simultaneously capturing high-resolution full well scans to allow for monitoring and trackability of each step of the iPSC manufacturing pipeline. Specifically, the Cell X platform consists of a syringe pump for picking and weeding of iPSC cultures (Fig. 1A^1^, B^1^), three peristaltic pumps (Fig. 1A^6^ and C) connected to three independent supply needles (Fig. 1B^3^ and D) for performing media exchanges, and a separate peristaltic pump connected to an independent aspiration line (Fig. 1A2 and B^2^) used for media aspiration. Both the syringe pump and aspiration pump can pick up and discard disposable micropipette tips to maintain aseptic cell culture conditions (Fig. 1E). This liquid handling platform is built over an automated inverted microscope with phase and fluorescent capabilities (Fig. 1A), which allows for the tracking of cultures from fibroblast isolation through iPSC colony formation and clonal expansion. For instance, using phase microscopy starting at 7 days post-fibroblast transduction, we were able to track iPSC colony formation over a 15-day time frame. (Fig. 1F–J).

Picking iPSCs for clonal expansion and weeding of residual somatic cells or zones of spontaneous differentiation are two of the most labor-intensive steps in modern iPSC generation protocols. After somatic cells isolated from a patient are transduced with reprogramming factors, the resulting culture must be maintained and monitored for several weeks before the reprogrammed iPSC colonies grow large enough to be selected (i.e., ~ 1.5-3 mm in diameter). During this period, the somatic cells from which the iPSCs were derived may need to be removed or `weeded` to prevent them from outcompeting the newly reprogrammed iPSCs. Once the iPSC colonies reach sufficient size, they must be harvested, or ‘picked’ and replated into individual cell culture vessels for clonal expansion. While the picked iPSCs are being clonally expanded, any regions that begin to spontaneously differentiate must be removed to maintain the integrity of the iPSC culture. For cell lines with excessive spontaneous differentiation, it may be necessary to re-pick the desired cells rather than weed the undesirable cells. Picking and weeding are typically done manually on a phase microscope using a micropipette. These tasks are time consuming and require highly skilled personnel, especially when performed under cGMP. Therefore, automating these procedures would remove a bottleneck in the therapy production pipeline which in turn would increase throughput. To create and validate standard operating procedures for automated iPSC generation, which includes picking, weeding and clonal expansion, dermal fibroblasts obtained from the 21 individuals listed in Fig. 1K were expanded and reprogrammed. This cohort was chosen as it had a wide age range (14 to 88 years with an average age of 50.2 years), included both 8 males and 13 females and represented normal individuals (i.e. ND = no disease) as well as patients with common (i.e., *ABCA4*-associated) and rare (i.e., *CRB1*-associated) forms of molecularly confirmed inherited retinal degeneration.

**Fig. 2:**
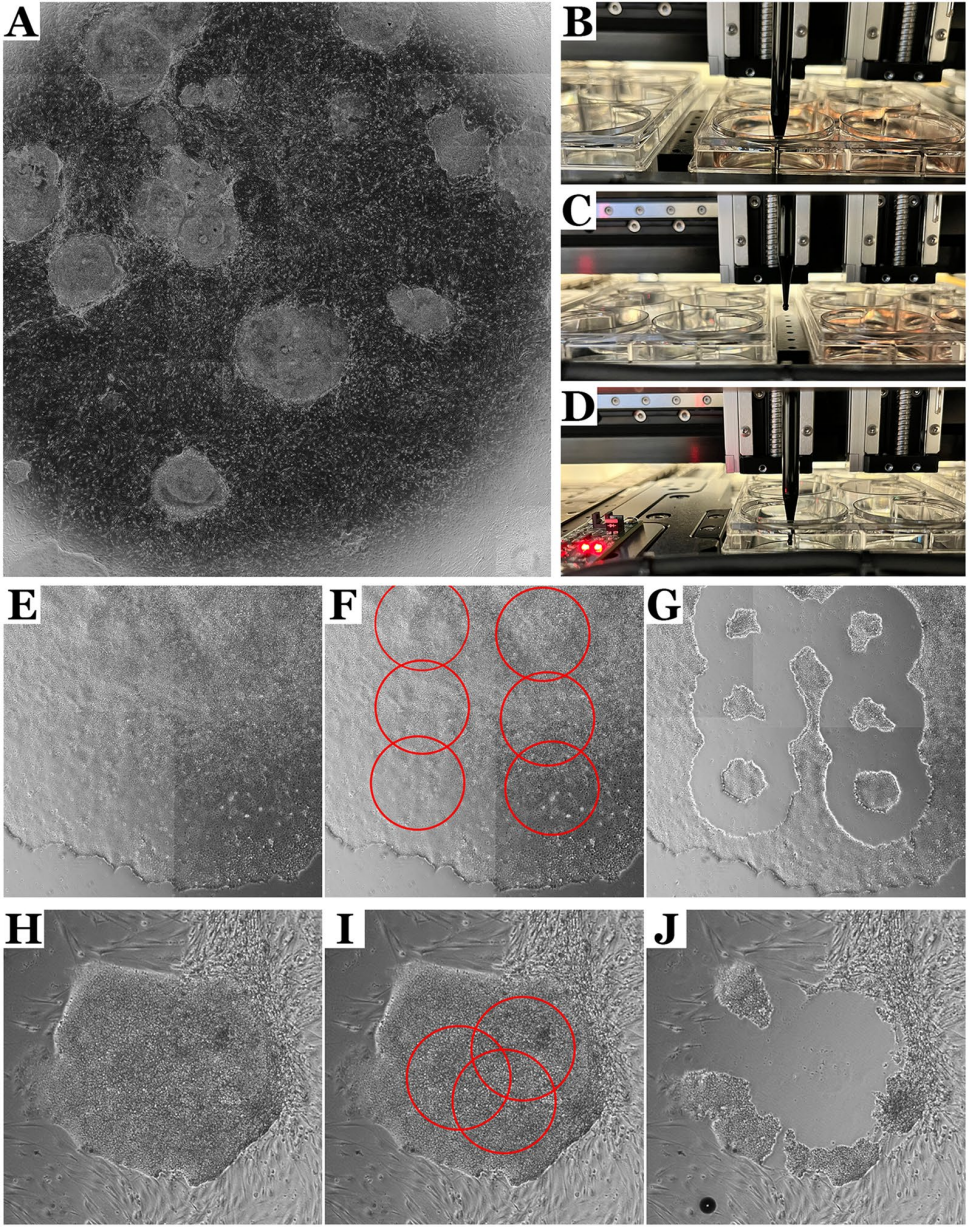
Automated picking of patient-derived iPSCs. **A** After reprogramming, the growth of iPSC colonies generated from primary dermal fibroblasts was monitored by using the Cell X platform to capture whole well scans each time the culture was fed. **B**–**D** Once the transformed iPSC colonies reached sufficient size, the Cell X platform’s robotic syringe pump was used to pick cells from areas selected by a user from the phase contrast montage of the donor well. The Cell X platform is capable of automatically mounting and removing disposable micropipette tips to maintain aseptic cell culture conditions. **E**–**G** the aspiration rates used to pick the iPSCs were optimized to find the lowest values of fluidic shear necessary to lift the cells. Given that the maximum shear stress occurs under the rim of the micropipette tip, cells are lifted from an annular region underneath this rim. **H**–**J** Overlapping pick points allow for picking of entire colonies without increasing the maximum applied fluidic shear

The Cell X platform is capable of robotically picking iPSC colonies using a syringe pump and sterile micropipette tip. To maintain cell viability and pluripotency, it is important to minimize the fluidic shear used to lift the cells. To optimize the pick parameters (tip height, aspiration volume and aspiration rate), iPSC cultures were picked at 25–30 days post-transduction (Fig. 2A). At this point, large dense colonies between 1.5 and 3 mm in diameter are present. As shown in Fig. 2, the picking process involves placing a sterile tip connected to the syringe pump into the donor plate above a colony and aspirating the desired volume of media at a defined rate (**B**) before moving the stage (**C**) from the donor plate to the desired well in the recipient plate where the sample is dispensed into a well containing culture media (**D**). The outer diameter of the picking pipette tip is 733 μm and the number of picks required to select an entire colony is determined by colony size. To accelerate the picking process, when picking from a single colony, several picks can be made prior to moving from the donor plate to the recipient plate. After an entire colony has been picked, the micropipette tip is replaced prior to picking the next colony.

As shown in Fig. 2E-G, given that the region of maximum fluidic shear corresponds to the area under the rim of the micropipette tip, the Cell X picking function collects cells in a distinctive annular region. While complete circles could be picked by increasing the syringe pump’s aspiration rate, this would increase the maximum applied shear beyond what is necessary, placing undue stress on the cells being collected. Instead, we chose to collect complete colonies by overlapping several pick points (Fig. 2H–J), which allows for optimal picking precision and increased cell survival. When using this function to weed unwanted spontaneous differentiation and remove somatic cells early in the reprogramming process, the aspiration speed and volume can be increased to allow complete removal.

### Validation of iPSCs generated using the cell X platform

**Fig. 3:**
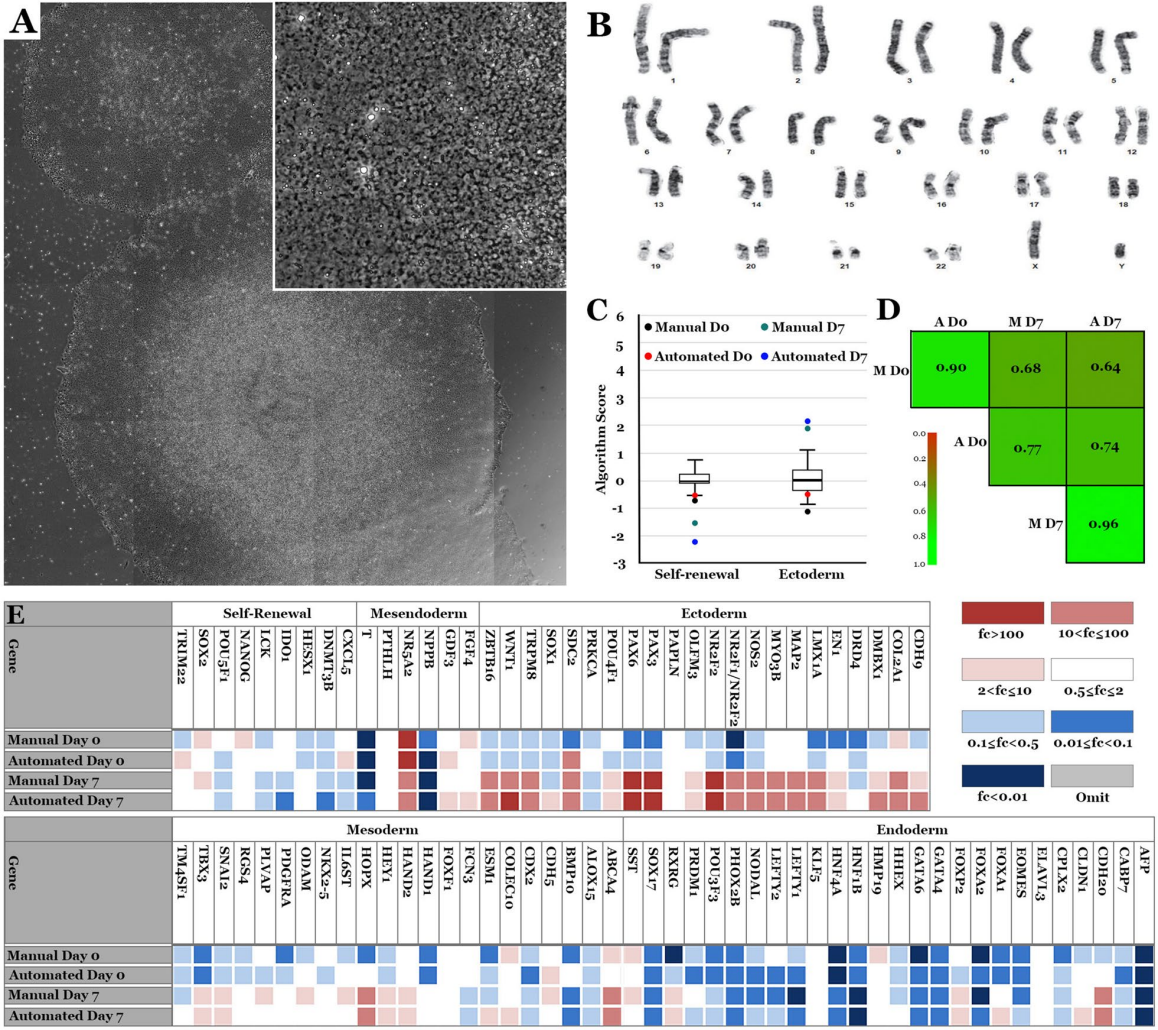
Validation of iPSCs generated on the Cell X platform. **A** A representative Cell X phase micrograph of iPSCs generated on the Cell X platform. **B** A representative karyotype of B1427 iPSCs. **C–E** TaqMan hPSC Scorecard analysis comparing iPSCs generated by manual or automated processing and their day 7 EBs. **C** Graph comparing the algorithm scores for expression of genes involved in self-renewal or ectoderm lineage. The undifferentiated reference set is indicated by the black box plot. **D** Correlation coefficients (r^2^ values). **E** Expression plots showing fold change in expression of the given genes compared to the undifferentiated reference set

**Fig. 4:**
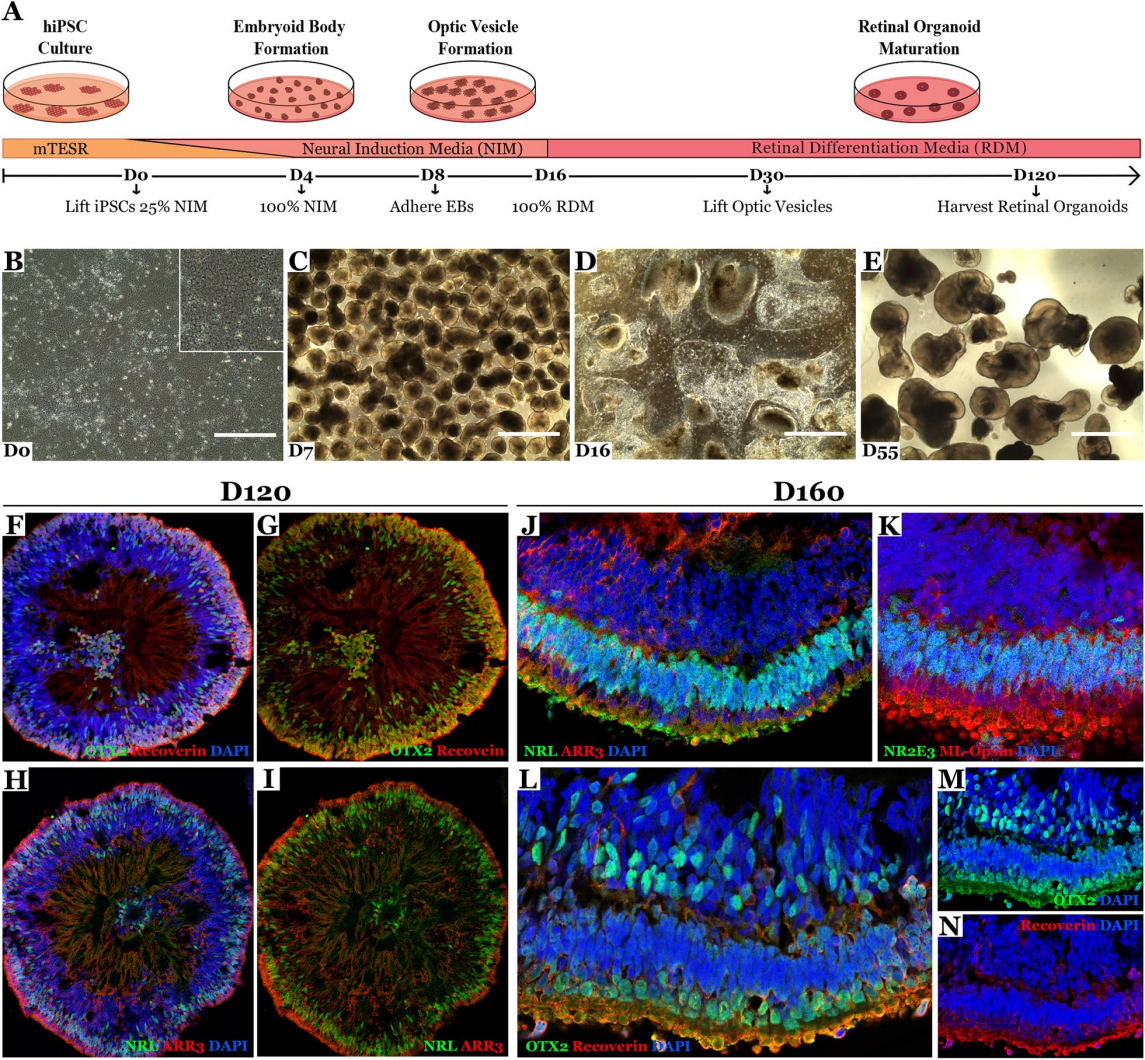
Retinal differentiation of iPSCs generated on the Cell X platform. **A** Schematic of retinal differentiation protocol. Representative phase micrograph of day 0 iPSCs (**B**), day 7 EBs (**C**), day 16 attached optic vesicles (**D**), and day 55 lifted retinal organoids. Scale bar = 1 mm. Immunohistochemical staining of retinal organoids at day 120 (**F**– **I**) and day 160 (**J–N**). Antibodies targeted the photoreceptor cell markers OTX2 (green) and Recoverin (red), the rod photoreceptor cell markers NRL and NR2E3 (green) and cone photoreceptor cell marker ARR3 and ML-Opsin (red). DAPI (blue) was used as a nuclear counterstain

IPSCs generated using the Cell X platform had normal iPSC morphology and karyotype (20 cells were analyzed per line) (Fig. 3A, B). TaqMan hPSC Scorecard analysis showed iPSCs generated using the Cell X platform (automated) had similar pluripotency and day 7 ectoderm expression to iPSCs generated manually (Fig. 3C, D, E). Next, we modified previously published retinal differentiation protocols [31, 50] to be cGMP-compliant (Fig. 4A) and iPSCs (B1427) were differentiated in the Biospherix Xvivo system. Cells showed normal morphology at different stages of retinal differentiation: iPSC (Fig. 4B), day 7 embryoid bodies (EBs) (Fig. 4C), day 16 attached optic vesicles (Fig. 4D), and lifted retinal organoids (Fig. 4E). Organoids analyzed on day 120 or day 160 expressed the photoreceptor markers OTX2, Recoverin, NRL, NR2E3, ARR3 and ML-Opsin (Fig. 4F–N). Fibroblasts from the same biopsy (B1427) were reprogramed manually and showed similar morphology and staining after different stages of retinal differentiation (Additional file 1: Fig. S1).

### Single-cell investigation of the cellular composition of retinal organoids

**Fig. 5:**
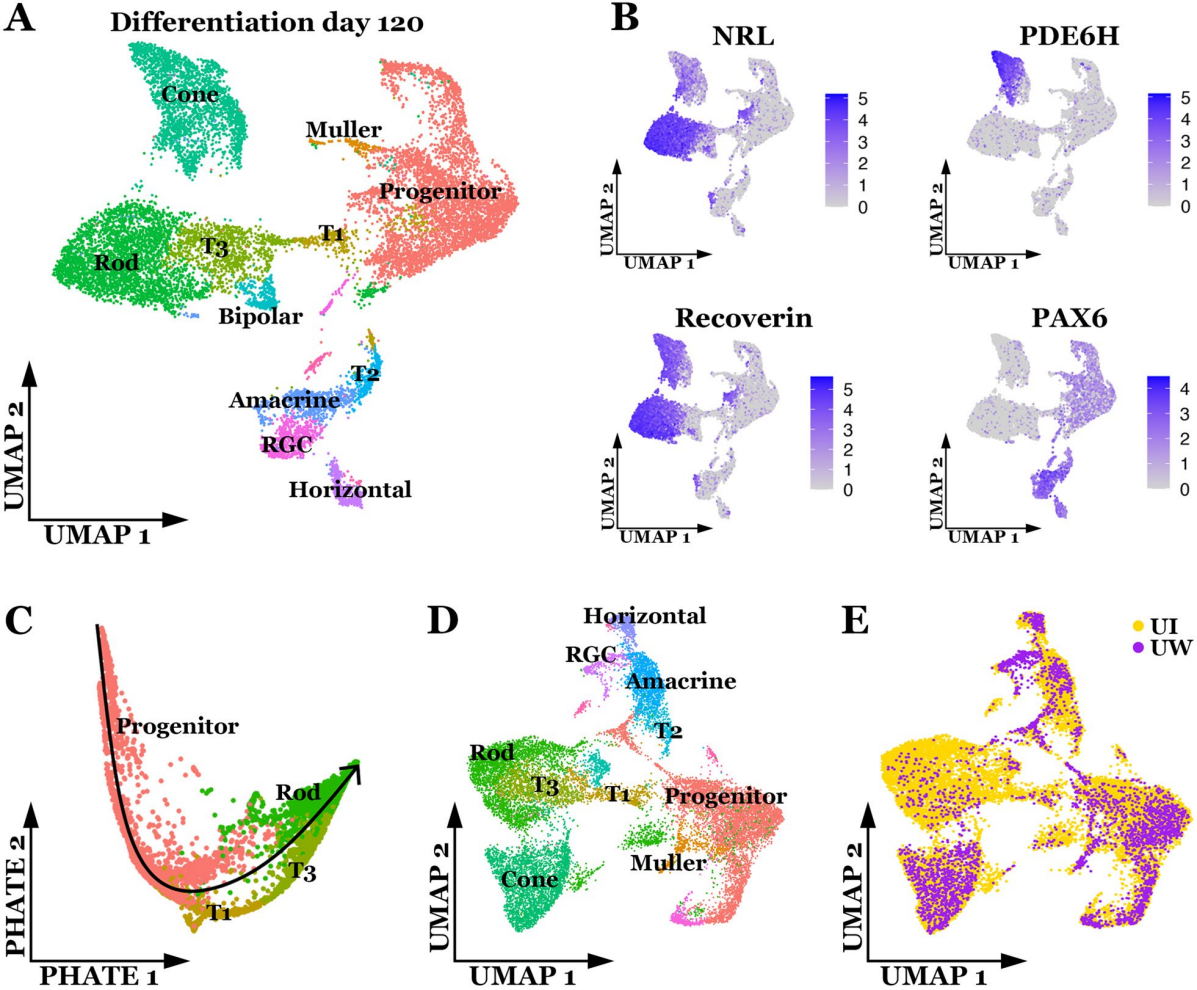
Gene expression in single cells from retinal organoids. **A** Two-dimensional (uniform manifold approximation and projection) UMAP embedding of organoid-derived cells shows presence of distinct neural retina cell types at day 120 of differentiation. **B** Expression of cell type-specific marker genes is shown based on shading of individual cells. Rod photoreceptors express *NRL*, cone photoreceptors express *PDE6H*, all photoreceptors express *RCVRN*, and progenitor and inner retinal populations express *PAX6*. **C** Two-dimensional PHATE embedding of cells in the rod lineage demonstrates organoid-derived cells across different stages of differentiation. **D** Two-dimensional UMAP embedding of organoid-derived cells from this study integrated with those from a previous study [52]. **E** Cells in D colored by study of origin. UI = cells from the current study (generated at University of Iowa), UW = cells from a previous study ( [52] generated at University of Washington). The cells generated via the method described in the current study integrate efficiently with those described previously

To investigate the cellular composition and maturation of organoids differentiated from iPSCs generated on the Cell X platform, single-cell RNA sequencing was used. Single cells were recovered from retinal organoids at differentiation day 120 (D120) and annotated based on gene expression profiles (Fig. 5A). All major classes of neural retinal cells were recovered, in addition to the progenitor and transitional populations described previously [52]. Expression of known photoreceptor marker genes was confirmed (Fig. 5B), where rod and cone photoreceptor types could be differentiated by *NRL* and *PDE6H* expression respectively. We next examined the rod differentiation lineage by subsetting only cells in the Progenitor, T1/T3 (transitioning progenitor populations developing towards photoreceptor lineages [52], and Rod clusters. These cells were visualized in two-dimensions using PHATE, a method designed to preserve global structure and cell state transitions [53] (Fig. 5C). A clear trajectory through expected cell types was observed, showing that organoid-derived cells in our system follow known developmental lineages. We next integrated our data with single-cell RNA sequencing data of retinal organoids generated by Sridhar et al. [52]. Dimensionality reduction was performed on the new integrated dataset and cells were annotated as described above (Fig. 5D). Every cluster in the resulting integrated data set contained cells from the current study as well as those from Sridhar et al. (Fig. 5D), indicating similarity in the transcriptional profiles of retinal cells generated from iPSCs produced using the Cell X platform and those generated manually in a different laboratory.

## Discussion

The discovery that pluripotent stem cells could be generated from dermal fibroblasts via forced expression of the genes *c-MYC*, *OCT-3/4*, *KLF4*, *SOX2* [55], revolutionized the field of regenerative medicine. Prior to this, pluripotent ESCs were the most favorable cell type available to clinical cell replacement laboratories. Widespread use of human ESCs enabled development of highly effective differentiation protocols, including those designed for derivation of transplantable retinal progenitor and photoreceptor precursor cells [9, 10, 14, 15, 17, 28, 30, 36–39].

As ESCs are well suited to large scale clinical manufacturing, they remain a widely used cell source for clinical cell replacement despite their allogenic nature and likely need for lifelong immune suppression post-transplantation. Although patient-derived iPSCs reduce host-donor matching concerns, for autologous iPSC-mediated cell replacement to be viable, development of cGMP-compliant manufacturing technologies that enable parallel production of multiple cell lines by a single technician are needed. To this end, we report development of the Cell X platform, a robotic platform that can automate many of the labor-intensive steps that are difficult to perform manually in large numbers under cGMP. Specifically, imaging and tracking of cell lineage, cell culture media replacement, iPSC colony picking and weeding of spontaneous differentiation are all tasks that the Cell X platform has been designed to accomplish.

One of the greatest advantages of using a robotic cell culture platform, such as the Cell X platform, is the ability to perform tasks in the absence of a user. Specifically, once the commands have been provided, the Cell X platform can be trusted to perform a series of validated tasks, in the same way, day-in and day-out. Not only can this increase throughput, but it also reduces batch-to-batch product variability, allowing one to be confident that any biological differences seen between cell lines are unlikely to be due to human error, inconsistencies introduced by slight technical variations, or protocol drift. Prior to implementation of a robotic system into a manufacturing pipeline, standard operating procedures need to be developed and validated for the specific application. In this study, we were interested in developing a system with the ability to track patient dermal fibroblasts from isolation through reprogramming and clonal expansion. In addition, a major goal was to automate the process of iPSC picking, a task that is both time consuming and challenging for technical staff to perform manually, especially when in full cleanroom attire. The picking strategy we employed utilizes a syringe pump to aspirate and dispense selected iPSC colonies. As shown in Fig. 2, strategic placement of the pick ring allows for either picking of the entire colony or partial picks. The latter are very useful for sampling and analysis following CRISPR-mediated genome editing. In addition to its utility for isolating desired cell populations, the syringe pump can also be used to remove undesirable spontaneously differentiated cell types. In areas where spontaneous differentiation is minimal, the differentiated cells can be aspirated and dispensed as waste. When larger areas of spontaneous differentiation exist, the pipette tip attached to the syringe pump can be used to scrape away the undesirable cell types and the aspirator, which is controlled by an independent peristaltic pump, can remove the media and floating debris. Inclusion of three additional peristaltic pumps, each of which can be connected to an independent media reservoir, provides the user with the ability to incorporate a wash step and media refill allowing for complete removal of undesirable cell types. At later stages of iPSC expansion, where picking of iPSCs and weeding of spontaneous differentiation becomes infrequent, the third peristaltic pump can be used to deliver a nonenzymatic passaging solution, such as Versene, for large scale passage of clonally expanded cell lines.

Several robotic cell culture systems, ranging from small liquid handling devices [56, 57] to large stand-alone multifunction platforms [58–66], have been reported. For instance, the CompacT SelecT (CTST) platform developed by Tristan et al., at the National Center for Advancing Translational Sciences, is a large stand-alone unit that incorporates an incubator, microscope, cell counter, several peristaltic pumps, chilled media storage area, and robotic plate handling device [58]. Using this system, the authors demonstrated simultaneous culture of up to 90 human iPSC lines for extended passage without inducing karyotypic abnormalities or loss of potency [58]. In a recent publication, Elanzew and colleagues described a similar system that they termed the StemCellFactory [59]. Unlike the CTST, which functions as a stand-alone piece of equipment that could be placed in an existing manufacturing suite, the StemCellFactory is a modular unit containing more than 30 integrated instruments all of which are housed inside a large custom laminar flow hood resembling a cleanroom [59]. In addition to a dedicated liquid handling device, two cell culture incubators, an automated microscope, and a robotic plate mover, this system includes a commercially available single cell / colony picking platform (CellCelector™, Sartorius), which is used to enable hiPSC generation and expansion [59]. As described above, the Cell X platform was designed to perform several of the different tasks that are carried out by independent modules in the StemCellFactory: automated imaging, feeding, cell picking and weeding.

As the Cell X has a relatively small footprint it can be placed inside of a standard laminar flow hood, which can be easily placed in an existing clean room environment. In this study, we opted to place the Cell X platform inside a cGMP compliant Biospherix Xvivo system (ISO Class 5 system with real time monitoring and trackability of gases, temperature, humidity, VOCs and particles) to allow for greater control over atmospheric conditions. Specifically, the entire unit, which includes the laminar flow hood containing the Cell X platform, adjacent processing chamber housing cell culture media and passaging reagents, and the cell culture incubators, is maintained at 37 °C with 5% CO2 and the oxygen concentration required for the specific task being performed. Reduced oxygen tension has been shown to enhance the rate of iPSC reprogramming [67]. As reprogramming of somatic cells from elderly individuals can be difficult [68], the ability to reduce atmospheric oxygen tension can be extraordinarily useful. In this study, patient derived iPSCs were generated, picked, clonally expanded, and maintained at 10% oxygen tension. Once reprogramming was complete, the oxygen tension was raised to 20% for long term iPSC culture and 3D retinal differentiation. At later stages of differentiation, retinal organoids can become large and difficult to perfuse. To enhance retinal cell viability under extended culture conditions, elevated oxygen levels have been used. For instance, in one of the first protocols published, Nakano and colleagues reported culture of 3D retinal organoids under 40% oxygen tension [30], which is readily achievable using the combined Biospherix / Cell X platform reported here.

Unlike the CTST and StemCellFactory, which have robotic arms designed to transfer cell culture dishes from one station to the next, when using the Cell X, a technician must be present to manually transfer plates and consumables both on and off the device. To enable continuous manufacturing, a 6-axis collaborative robotic arm (Universal Robots, Odense, Denmark), high-capacity incubator (Liconic STX-500, Mauren, Liechtenstein) and custom master scheduling software are currently being incorporated. The 6-axis robotic arm will move plates of cells from the high-capacity incubator and consumables from their storage locations onto the Cell X platform to enable hands free iPSC generation, culture, and differentiation. The custom master scheduling software is being designed to allow a technician to set a series of tasks for each cell line from initiation of reprogramming through retinal differentiation, which can be modified as needed depending on real time data analysis. If when reviewing imaging data, it is determined that a subsequent step needs to be performed sooner or later than originally planned, the date can be modified and all downstream steps adjusted accordingly. For example, the iPSC generation protocol used in this study specifies that initial colonies be picked at approximately 25 days following Sendai viral transduction of patient dermal fibroblasts. If the original schedule was set to image and feed transduced cells daily with colony picking at day 25, yet on day 24 data review revealed that colonies were growing more slowly and had not yet reached the desired size, then picking could be delayed and the downstream clonal expansion, passaging and differentiation dates adjust accordingly.

## Conclusion

In this work, we have shown that the Cell X platform can be used to automate many of the time and labor-intensive steps required for iPSC generation. As it can image the entire cell culture surface at any frequency, it enables a level of trackability that is not possible using standard cell culture and imaging approaches. While protocol development is critical, once a Cell X compatible standard operating procedure has been validated, the platform can be used to complete tasks without deviation, resulting in exceptionally low batch-to-batch variation. Collectively, these features greatly increase both the production capability of a single technician and the quality of the product being developed, making it ideally suited for incorporation into a clinical production pipeline.

## Supplementary Information

**Additional file 1: Fig. S1. **Retinal differentiation of manually generated iPSCs. Representative phase micrograph of day 0 iPSCs (**A**), day 7 EBs (**B**), and day 40 lifted retinal organoids (**C**). Scale bar = 250 μm (**A**) and 1 mm (**B**, **C**). Immunohistochemical staining of retinal organoids at day 120 (**D**–**G**) and day 160 (**H**–**N**). Antibodies targeted the photoreceptor cell markers OTX2 (red) and Recoverin (green), the rod photoreceptor cell markers NRL (red) and NR2E3 (green) and cone photoreceptor cell marker ARR3 (green (**D**, **E**, **H**, **I**) and red (**L**, **N**)). DAPI (blue) was used as a nuclear counterstain

## Data Availability

The single-cell RNA expression data that support the findings presented in this study are reported in their entirety and will be available for public download (GEO # GSE220154). All other data are available in the article and online supplementary material.

## Abbreviations

PNS: Peripheral nervous system
CNS: Central nervous system
iPSC: Induced pluripotent stem cells
E8: Essential 8 medium
ESC: Embryonic stem cells
cGMP: Current good manufacturing practices
EB: Embryoid bodies
ND: No disease
scRNA-seq: Single-cell RNA sequencing
