# Supplementary figures and images for: Automating iPSC generation to enable autologous photoreceptor cell replacement therapy
Source: J Transl Med. 2023 Feb 28;21:161. doi: 10.1186/s12967-023-03966-2 (PMC9976478; doi:10.1186/s12967-023-03966-2)

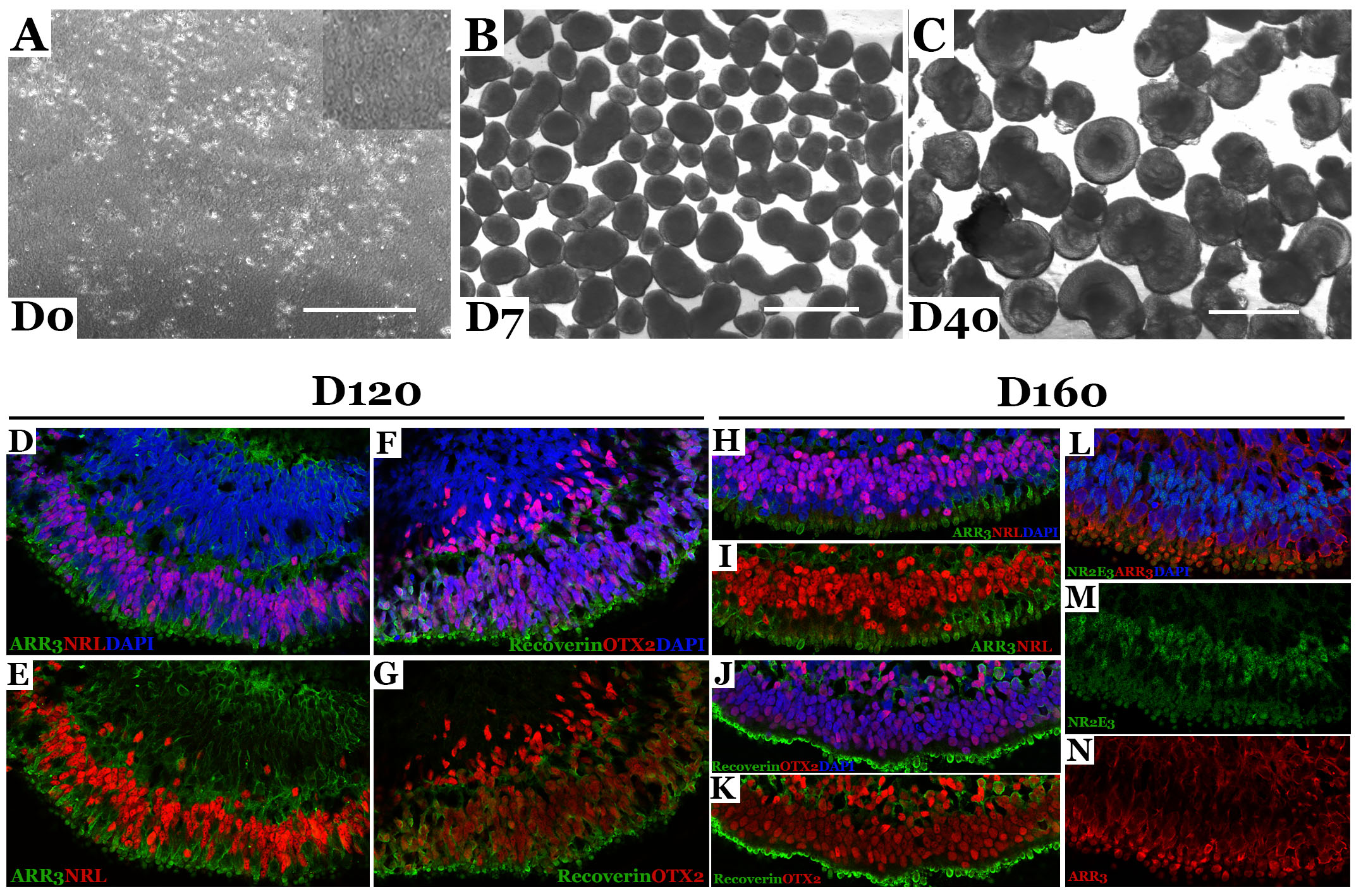

Supplement: Supplementary file 1 — Additional file 1: Fig. S1. Retinal differentiation of manually generated iPSCs. Representative phase micrograph of day 0 iPSCs (A), day 7 EBs (B), and day 40 lifted retinal organoids (C). Scale bar = 250 μm (A) and 1 mm (B, C). Immunohistochemical staining of retinal organoids at day 120 (D–G) and day 160 (H–N). Antibodies targeted the photoreceptor cell markers OTX2 (red) and Recoverin (green), the rod photoreceptor cell markers NRL (red) and NR2E3 (green) and cone photoreceptor cell marker ARR3 (green (D, E, H, I) and red (L, N)). DAPI (blue) was used as a nuclear counterstain [file 12967_2023_3966_MOESM1_ESM.jpg]
